# Traumatic Brain Injury and Genetic Risk for Alzheimer’s Disease Impact Cerebrospinal Fluid β-Amyloid Levels in Vietnam War Veterans

**DOI:** 10.1089/neur.2024.0048

**Published:** 2024-08-22

**Authors:** Jena N. Moody, Erica Howard, Kate E. Nolan, Sarah Prieto, Mark W. Logue, Jasmeet P. Hayes, for the Alzheimer’s Disease Neuroimaging Initiative

**Affiliations:** ^1^ Department of Psychology, The Ohio State University, Columbus, Ohio, USA.; ^2^ National Center for PTSD, VA Boston Healthcare System, Boston, Massachusetts, USA.; ^3^ Psychiatry and Biomedical Genetics, Boston University Chobanian & Avedisian School of Medicine, Boston, Massachusetts, USA.; ^4^ Department of Biostatistics, Boston University School of Public Health, Boston, Massachusetts, USA.; ^5^ Chronic Brain Injury Initiative, The Ohio State University, Columbus, Ohio, USA.

**Keywords:** traumatic brain injury, polygenic risk, Alzheimer’s disease, β-amyloid, Vietnam Veterans

## Abstract

Traumatic brain injuries (TBIs) may increase the risk for Alzheimer’s disease (AD) and its neuropathological correlates, although the mechanisms of this relationship are unclear. The current study examined the synergistic effects of TBI and genetic risk for AD on β-amyloid (Aβ) levels among Vietnam War Veterans. We hypothesized that the combination of TBI and higher polygenic risk score (PRS) for AD would be associated with lower cerebrospinal fluid (CSF) Aβ_42/40_. Data were obtained from the Department of Defense Alzheimer’s Disease Neuroimaging Initiative. Participants included Vietnam War Veterans without dementia who identified as White non-Hispanic/Latino and had available demographic, clinical assessment, genetic, and CSF biomarker data. Lifetime TBI history was assessed using The Ohio State University TBI Identification Method. Participants were categorized into those with and without TBI. Among those with a prior TBI, injury severity was defined as either mild or moderate/severe. CSF Aβ_42/40_ ratios were calculated. Genetic propensity for AD was assessed using PRSs. Hierarchical linear regression models examined the interactive effects of TBI and PRS for AD on Aβ_42/40_. Exploratory analyses examined the interaction between TBI severity and PRS. The final sample included 88 male Vietnam War Veterans who identified as White non-Hispanic/Latino (*M*_age_ = 68.3 years), 49 of whom reported a prior TBI. There was a significant interaction between TBI and PRS, such that individuals with TBI and higher PRS for AD had lower Aβ_42/40_ (*B* = −0.45, 95% CI: −0.86 to −0.05, *p* = 0.03). This relationship may be stronger with increasing TBI severity (*p* = 0.05). Overall, TBI was associated with lower Aβ_42/40_, indicating greater amyloid deposition in the brain, in the context of greater polygenic risk for AD. These findings highlight *who* may be at increased risk for AD neuropathology following TBI.

## Introduction

Traumatic brain injury (TBI) results from a force to the head that leads to clinical symptoms such as loss of consciousness, post-traumatic amnesia, or altered mental state. TBI is associated with chronic neural and cognitive alterations^1,2^ along with immense societal and economic costs.^3^ A foremost concern is the mounting evidence that TBI confers greater risk for dementia, including Alzheimer’s disease (AD),^4–7^ and that the age of dementia onset is earlier.^8^ Despite the substantial evidence and continued interest in the connection between TBI and AD, the exact mechanisms by which TBI amplifies the risk for AD remain unclear.

A key neuropathological AD marker is extracellular β-amyloid (Aβ) neuritic plaque accumulation. In AD, neuritic plaques form via altered cleaving of the amyloid precursor protein (APP). APP alterations result in the aggregation of Aβ fibrils that form plaques, and oligomers thought to induce neurotoxicity and promote formation of tau protein neurofibrillary tangles.^9^ *In vivo* measurement of Aβ shows that as plaque deposits increase, cerebrospinal fluid (CSF) Aβ decreases.^10^ Studies of TBI brains at autopsy provide evidence for aberrant APP processing,^11,12^ raising the possibility that increased risk for AD following TBI is related to exacerbation of the primary neuropathology of AD. However, existing research on the link between TBI and CSF Aβ has yielded varied results, with some studies reporting reduced CSF Aβ_42_ levels in individuals with severe TBI,^13^ whereas others found no notable differences in CSF Aβ levels between individuals with and without head impacts.^14,15^ Importantly, there is an emerging recognition that genetic risk factors moderate the association between TBI and markers of neurodegeneration, potentially resolving the observed discrepancies in findings.

The apolipoprotein E (*APOE*) ε4 allele is undoubtedly the strongest individual genetic risk factor for AD, although polygenic approaches can explain incremental variance in the AD phenotype by considering the relatively small effects of additional genetic variants that confer risk for AD.^16,17^ Prior studies have demonstrated that the relationship between TBI and AD varies as a function of genetic risk, such that there is increased AD risk and neuropathology following TBI among *APOE* ε4 carriers^18–20^ and individuals with higher polygenic risk score (PRS) for AD,^21^ which may be driven by impaired Aβ clearance.^22–24^ However, additional research is needed to determine the moderating role of genetic susceptibility for AD on the relationship between TBI and CSF Aβ levels. This line of work is of paramount importance, not only for the identification of early risk for AD but also to provide a window into in vivo preclinical pathophysiological processes following TBI.

The current study aimed to examine relationships between TBI, genetic risk for AD, and CSF Aβ among Vietnam War Veterans without dementia. We measured the Aβ_42/40_ ratio in CSF, which may better capture brain amyloid accumulation in prodromal AD than individual Aβ isoforms.^25^ In addition, given that the relationship between TBI and AD may differ as a function of injury severity (i.e., moderate/severe versus mild),^26,27^ we explored the interaction between TBI severity and genetic risk for AD. We hypothesized that the combination of TBI and higher AD PRS would be associated with lower CSF Aβ_42/40_, reflecting greater AD pathology, and that greater TBI severity would be associated with lower CSF Aβ_42/40_.

## Materials and Methods

### Participants

Data were obtained from the Department of Defense Alzheimer’s Disease Neuroimaging Initiative (DOD-ADNI; adni.loni.usc.edu), a publicly available database with neuroimaging, genetic, biomarker, and clinical/cognitive data. DOD-ADNI is a multicenter project that examines the long-term effects of TBI and post-traumatic stress disorder (PTSD) among Vietnam War Veterans and determines how these factors may be related to AD. Details about DOD-ADNI are described elsewhere.^28,29^ Study procedures were approved by site-specific Institutional Review Boards (IRBs), and all participants provided written informed consent consistent with the Declaration of Helsinki. Given that this study solely analyzes publicly available data, a local IRB determined the current analyses are exempt from IRB approval and participant consent was waived due to the retrospective nature of the study and previously provided written informed consent to enroll in the DOD-ADNI database (The Ohio State University Human Research Protection Program study number 2021E1143).

The final sample included 88 male Veterans who identified as White non-Hispanic/Latino and were enrolled in DOD-ADNI. First, we calculated PRSs for individuals who identified as White non-Hispanic/Latino to avoid population stratification effects (*n* = 162). We excluded 12 participants who were missing clinical assessment data and 62 participants who were missing CSF biomarker data. Individuals were excluded from DOD-ADNI if they had dementia, clinical evidence of stroke, psychosis or bipolar disorder, alcohol/substance abuse/dependence, a seizure disorder, or unstable major medical conditions (e.g., cancer and cardiovascular disease). Additional information is available online in the DOD-ADNI protocol (https://adni.loni.usc.edu/wp-content/uploads/2013/09/DOD-ADNI-IRB-Approved-Final-protocol-08072012.pdf).

### Clinical assessment

Lifetime TBI history was assessed using The Ohio State University TBI Identification Method—Interview Form, a psychometrically validated structured interview that is currently the gold standard for retrospectively assessing lifetime TBI history.^30^ During the interview, participants were asked about injuries to their head/neck that occurred before, during, and after Vietnam. TBI severity was classified based on the VA/DOD criteria (Table 1).^31^

**Table 1.** Classification of TBI severity

	Mild	Moderate/Severe
Loss of consciousness	<30 minutes	≥30 minutes
Post-traumatic amnesia	≤24 hours	>24 hours
Altered mental state	≤24 hours	>24 hours

TBI severity was classified based on the VA/DOD criteria.^31^ If more than one severity category was met, the higher severity (i.e., moderate/severe TBI) was assigned.

TBI, traumatic brain injury.

Lifetime PTSD symptoms were assessed using the Clinician Administered PTSD Scale (CAPS-IV),^32^ which is the gold standard PTSD assessment that provides a categorical diagnosis and continuous severity score. During the interview, participants were asked about the frequency and intensity of PTSD symptoms. The present study analyzed the CAPS-IV severity score, as it better reflects individual variability in PTSD symptoms than a dichotomous diagnostic variable.

### Genotyping and PRS computation

*APOE* and genome-wide genotyping were completed by DOD-ADNI using DNA from a blood sample. Genome-wide genetic data were generated with the Illumina HumanOmniExpress BeadChip and processed with GenomeStudio v2009.1 (Illumina). Additional information about genotyping procedures is available elsewhere.^33^

Using the genotype data provided by DOD-ADNI, we performed imputation using Eagle v2.4/Minimac4 on the Michigan Imputation Server^34^ (https://imputationserver.sph.umich.edu) with 1000 genomes Phase 3 reference data (apps@1000g-phase-3-v5, hg19).^35^ Next, we computed genome-wide PRSs to denote genetic propensity for AD in a single score. Specifically, all single nucleotide polymorphisms (SNPs) under a particular *p* value threshold were weighted using beta values from a genome-wide association study (GWAS),^36^ then multiplied by the additively coded genotypes (i.e., 0, 1, 2) and summed together. PRSs are typically calculated across various *p*-value thresholds, as there is no *a priori* way to determine the most predictive threshold. Consistent with our prior work,^21^ we calculated PRSs across six *p* value thresholds: *p* < 0.05, *p* < 0.10, *p* < 0.20, *p* < 0.30, *p* < 0.40, and *p* < 0.50. PRSs were calculated from hard-call genotype data generated from imputed genotypes with a call threshold of .8. Rare alleles (minor allele frequency < 0.01) and alleles with high missing data rates (> 0.05) were excluded from the calculation. PRSs were calculated using PRSice v2.3.3 for the Mac operating system^37^ based on summary data from the International Genomics of Alzheimer’s Disease Project GWAS (https://www.niagads.org/datasets/ng00075).^36^ To determine the degree to which PRSs are influenced by the *APOE* locus, AD PRSs were calculated first from the whole genome and then again excluding the *APOE* risk locus. As linkage disequilibrium (non-independent inheritance of closely spaced genomic variants) is strong in the region of *APOE*, the region from chr19 44,409,039 bp to 46,412,650 bp (GRCh37/hg19) was excluded.

### CSF biomarkers

CSF levels of Aβ_1-42_ and Aβ_1-40_ were analyzed using Elecsys electrochemiluminescence immunoassays on a fully automated cobas e601 platform (Roche Diagnostics).^38^ Samples were analyzed using a single lot of reagents for each biomarker. CSF samples were processed in batches. For quality control and to track longitudinal performance of the assay, a pooled CSF sample was processed with each batch. There were 26 participants with Aβ_1-42_ values greater than the upper technical limit of assay (1700 pg/mL), for whom Aβ_1-42_ values were extrapolated based on the calibration curve.^39^ There were no values below the lower technical limit for Aβ_1-42_ (200 pg/mL) or outside the technical limits for Aβ_1-40_ (11–43,000 pg/mL). Aβ_42/40_ ratio was calculated by DOD-ADNI by dividing Aβ_1-42_ by Aβ_1-40_.

### Statistical approach

Statistical analyses were performed using R version 4.1.1 for Macintosh. Aβ_42/40_ ratios and PRSs were standardized (*M* = 0, *SD* = 1) for analyses to aid interpretation. Participants with and without TBI were compared on demographic and outcome variables using *t*-tests for continuous variables and Fisher’s exact tests for categorical variables. Hierarchical linear regression models examined the main and interactive effects of TBI and genetic risk for AD on Aβ_42/40_ across all six PRS *p*-value thresholds. Covariates for all models and follow-up tests included age, education,^40^ and CAPS-IV severity score.^29,41^ Main effects are reported from models including all covariates and the main effects of TBI and PRS. Interaction effects are reported from models including all covariates, main effects, and the TBI by PRS interaction term. As the AD PRSs are highly correlated across the different *p* value thresholds (pairwise *r* = 0.85 to 0.99), a Bonferroni correction for six thresholds examined would be very conservative. Therefore, to impose strict multiple-testing comparison across the six PRS *p* value thresholds, Monte-Carlo null simulation with 10,000 replicates was used.^42^ This simulation randomly permutes the genetic data between participants and accounts for the correlations between the six PRS thresholds. The percentile of the observed *p* value in the minimum *p* value distribution (across the six thresholds) was taken as the multiple-testing corrected *p* value.^42^ Follow-up analyses were performed with the PRS threshold that had the strongest interaction with TBI on Aβ_42/40_, which was at the *p* < 0.50 threshold (see Supplementary table S1 for results at each *p* value threshold). This threshold was used in all further analyses.

*Post hoc* analyses were implemented to further understand the effects of PRS, TBI, and covariates on Aβ_42/40_. Given that there is often a dose–response relationship between TBI severity and long-term sequela,^26,27^ we ran an exploratory ANCOVA to examine the interaction between TBI severity and PRS. In addition, follow-up linear regressions explored the influence of *APOE* ε4 on the observed relationship of TBI and Aβ_42/40_ by examining the interaction between TBI and 1) AD PRS that excludes the variants in the *APOE* region and 2) *APOE* ε4 carrier status (i.e., 0 versus 1+ ε4 alleles).

## Results

### Sample characteristics

The final sample included 88 male Vietnam War Veterans, with a mean age of 68.3 (3.60) years, who identified as White non-Hispanic/Latino. Demographics of the entire cohort and demographics stratified by TBI status are presented in Table 2. There were no significant differences between those with and without TBI in terms of age, education, CAPS-IV severity score, PRS, *APOE* ε4 carrier status, or Aβ_42/40_ (all *p*’s > 0.27, see Table 2). Among the 49 participants with TBI, 23 participants had mild TBI, 24 participants had moderate/severe TBI, and for two participants, injury severity could not be determined due to discrepant information regarding TBI history; these two participants were excluded from TBI severity analyses. Moreover, 27 participants reported one prior injury, 20 participants reported more than one prior injury, and number of injuries could not be calculated for two participants due to discrepant information regarding TBI history. Demographics as a function of most severe TBI are presented in Supplementary table S2.

**Table 2.** Demographics of the overall sample, participants without TBI, and participants with TBI

	Total (*N* = 88)	No-TBI (*n* = 39)	TBI (*n* = 49)	Statistics
Age in years	68.3 (3.60)	67.8 (3.77)	68.6 (3.45)	*t*(78.02) = −1.09, *p* = 0.28
Education in years	15.1 (2.39)	14.8 (2.25)	15.3 (2.51)	*t*(84.71) = −0.93, *p* = 0.36
CAPS-IV severity score	46.1 (34.8)	45.8 (41.2)	46.3 (29.1)	*t*(65.93) = −0.06, *p* = 0.95
Polygenic risk score^a^	0.00 (1.00)	0.08 (1.02)	−0.06 (0.99)	*t*(80.30) = 0.67, *p* = 0.50
*APOE* ε4 status, *n* (%)^b^				OR = 1.24, *p* = 0.81^c^
0	65 (74%)	30 (77%)	35 (71%)	
1	22 (25%)	9 (23%)	13 (27%)	
Aβ_42/40_^a^	0.00 (1.00)	0.10 (0.96)	−0.08 (1.03)	*t*(83.99) = 0.87, *p* = 0.39

Values presented are mean (SD) unless otherwise indicated. Statistics reported are from t-tests unless otherwise indicated.

^a^ Standardized value.

^b^ One participant with a TBI was missing *APOE* ε4 status data.

^c^ Statistics reported from Fisher’s exact test.

TBI, traumatic brain injury.

### TBI and AD PRS on Aβ_42/40_

**Fig. 1.**
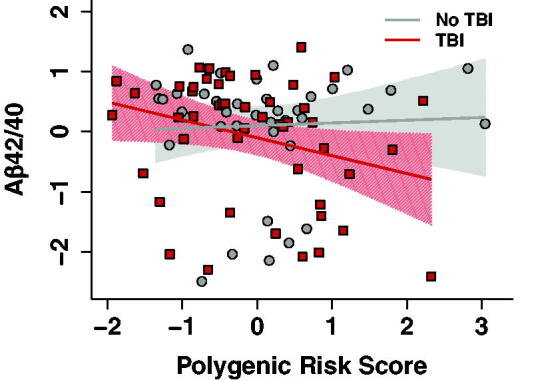
Lower Aβ_42/40_ among individuals with TBI and higher AD PRS. Results from a TBI × AD PRS interaction model. In the TBI group (red line/squares), higher AD PRS was associated with lower CSF Aβ_42/40_, suggesting greater Aβ pathology with increasing genetic risk for AD. This relationship was absent in the no TBI group (gray line/circles). Values on the x-axis represent AD PRS, with higher values indicating increased genetic risk for AD. Values on the y-axis represent Aβ_42/40_, with lower values indicating greater pathology. Shading represents 95% CIs. AD PRS and Aβ_42/40_ were standardized. AD, Alzheimer’s disease; CSF, cerebrospinal fluid; PRS, polygenic risk score; TBI, traumatic brain injuries.

There were no significant main effects of TBI (*B* = −0.19, 95% CI: −0.60 to 0.22, *p* = 0.36) or PRS (*B* = −0.12, 95% CI −0.33 to 0.09, *p* = 0.27) on Aβ_42/40_. There was, however, a significant interaction between TBI and PRS such that individuals with TBI and higher polygenic risk for AD had lower CSF Aβ_42/40_ (*B* = −0.45, 95% CI: −0.86 to −0.05, *P*_uncorrected_ = 0.03, *P*_corrected_ = 0.0495; Table 3 and Figure 1). To parse this interaction, partial correlations were used to examine the relationship between PRS and Aβ_42/40_ for TBI and no-TBI groups. After adjusting for all covariates, higher PRS was correlated with lower Aβ_42/40_ in the TBI group (*B* = −0.33, *p* = 0.02). There was no correlation between the PRS and Aβ_42/40_ in the no-TBI group (*B* = 0.17, *p* = 0.31).

**Table 3.** Summary of regression analysis for association with Aβ_42/40_

Variable	Model 1			Model 2			Model 3		
	B	SE (B)	p	B	SE (B)	p	B	SE (B)	p
Age	0.01	0.03	0.70	0.01	0.03	0.71	0.01	0.03	0.67
Education	−0.05	0.05	0.33	−0.05	0.05	0.27	−0.07	0.05	0.15
CAPS-IV Score	0.01	0.003	0.003^*^	0.01	0.003	0.007^*^	0.01	0.003	0.004^*^
TBI				−0.19	0.21	0.36	−0.18	0.20	0.38
PRS				−0.12	0.11	0.27	0.12	0.15	0.41
TBI × PRS							−0.45	0.20	0.03^a^^,^^*^
R^2^		0.122			0.143			0.192	
Model *F*		3.872^*^			2.725^*^			3.207^*^	

Polygenic risk and Aβ_42/40_ were standardized for analyses. The main effects of TBI and PRS are reported from model 2. The interaction between TBI and PRS is reported from model 3.

^a^ Corrected value of this interaction term = 0.0495 at the PRS threshold *p* < 0.50.

^*^ *p* < 0.05

PRS, polygenic risk score; TBI, traumatic brain injury.

**Fig. 2.**
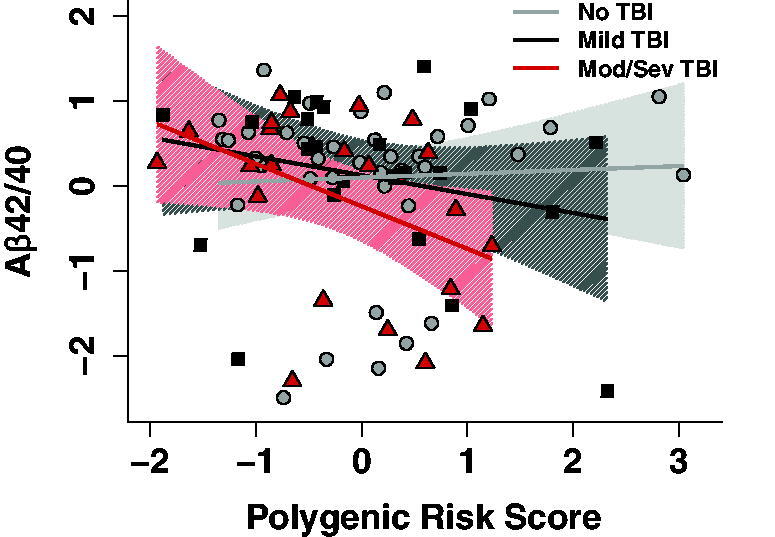
Relationship between Aβ_42/40_ and AD PRS as a function of TBI severity. Results from a TBI severity × AD PRS interaction model. A marginal interaction was observed suggesting a stronger relationship between AD PRS and CSF Aβ_42/40_ with increasing TBI severity. Values on the x-axis represent AD PRS, with higher values indicating increased genetic risk for AD. Values on the y-axis represent Aβ_42/40_, with lower values indicating greater pathology. Shading represents 95% CIs. Triangles represent individuals with a moderate/severe TBI history, squares represent individuals with a mild TBI history, and circles represent individuals with no TBI history. AD PRS and Aβ_42/40_ were standardized. AD, Alzheimer’s disease; CSF, cerebrospinal fluid; PRS, polygenic risk score; TBI, traumatic brain injuries.

When considering TBI severity (no-TBI, mild TBI, moderate/severe TBI), the ANCOVA model showed that the interaction between TBI severity and PRS was marginally significant in the expected direction (*p* = 0.05). Specifically, the relationship between polygenic risk and Aβ_42/40_ may be stronger with increasing TBI severity (Figure 2 and Supplementary table S3).

### TBI and *APOE* on Aβ_42/40_

To examine the possibility that *APOE* variants were the primary drivers of the PRS effect, we calculated a PRS that excluded variants in the *APOE* region. As with the PRS with *APOE* variants, there was no main effect of PRS on Aβ_42/40_ when variants in the *APOE* region were excluded from the score (*B* = −0.10, 95% CI: −0.31 to 0.12, *p* = 0.38). However, there was a significant TBI by PRS interaction (*B* = −0.46, 95% CI: −0.87 to −0.05, *p* = 0.03; Supplementary table S4), demonstrating that the interaction on Aβ_42/40_ remains with or without *APOE* included in the PRS calculation. Notably, there was a significant main effect of *APOE* ε4 carrier status, such that having at least one *APOE* ε4 allele was associated with lower Aβ_42/40_ (*B* = −1.12, 95% CI: −1.55 to −0.69, *p* < 0.001). However, there was no significant interaction between TBI and *APOE* ε4 carrier status on Aβ_42/40_ (*B* = −0.28, 95% CI: −1.14 to 0.57, *p* = 0.51; Supplementary table S5).

## Discussion

This study explored associations between TBI and CSF Aβ_42/40_ among male Vietnam War Veterans who were characterized by polygenic risk for AD. There were several notable findings. First, although there were no independent effects of TBI and PRS, there was an interaction between TBI and AD PRS on CSF Aβ_42/40_ levels, whereby having a TBI along with higher genetic risk for AD was associated with lower Aβ_42/40_. *APOE* ε4 carrier status strongly predicted lower CSF Aβ_42/40_ but *APOE* ε4 did not interact with TBI. In addition, the results suggested a dose–response relationship between TBI severity, PRS, and CSF Aβ_42/40_, such that the relationship may be stronger with more severe injuries (i.e., moderate/severe versus mild). Findings in this study should be considered provisional until the study can be replicated with larger sample sizes.

Epidemiological studies^4,5,8^ and meta-analyses^6,7^ demonstrate that TBI increases the risk for dementia. However, others find no association between TBI and dementia,^29,43^ highlighting the complexity of this relationship. Findings from this study highlight that genetic risk for AD moderates TBI in relation to Aβ dynamics, offering one potential explanation for prior inconsistent results. Specifically, individuals with a higher genetic predisposition for AD exhibit a reduction in CSF Aβ_42/40_ post-TBI compared with those without such genetic markers. This finding underscores the potential for a synergistic interaction between genetic vulnerability and TBI, whereby the combined effect confers greater risk for AD pathology, specifically Aβ. In addition, the findings suggest that TBI, in the context of polygenic risk for AD, may implicate biological pathways beyond *APOE* ε4 alone to increase Aβ plaque accumulation. Genetic pathways of AD include clathrin-mediated endocytosis, inflammation, oxidative stress, and immune response.^44,45^ Importantly, clathrin-mediated endocytosis plays a key role in APP processing and consequently Aβ plaque accumulation.^46^ Similarly, TBIs are associated with altered APP processing, inflammation, oxidative stress, and immune response.^11,12,47,48^ The substantial overlap in these neurometabolic pathways suggests that TBI may exacerbate the effects of genetic pathways associated with AD to increase neuropathology, notably Aβ plaque accumulation, although future research should investigate the specific mechanisms underlying this interaction. In particular, future work should use pathway-specific PRS scores^36,49^ to explicate the specific genetic pathways that may be implicated by TBI to contribute to increased Aβ pathology, which we were underpowered to explore here. The diminished CSF Aβ_42/40_ in genetically predisposed individuals post-TBI not only highlights a potential biomarker for early AD detection but also emphasizes the necessity of personalized therapeutic approaches that take into account an individual’s genetic landscape. Nonetheless, it is imperative to consider that while Aβ pathology is associated with risk for AD, it does not necessarily indicate that an individual will develop AD or the severity of cognitive decline, as there may be protective factors that promote cognitive and functional resilience in the context of Aβ deposition.^50^

The relationship between TBI and AD PRS on CSF Aβ may be stronger as a function of increasing injury severity. Although this interaction did not quite reach statistical significance, this is likely due to small sample sizes. These analyses should be replicated with larger sample sizes, and current findings should be considered exploratory. Nonetheless, this pattern of results is consistent with previous work demonstrating that more severe TBIs are associated with more long-term deficits^51^ and greater risk for AD.^26,27^ More severe injuries induce greater neurometabolic disruptions,^52^ including aberrant APP processing,^53^ suggesting that more severe injuries may exacerbate the genetic pathways of AD more than milder injuries, consequently leading to greater Aβ plaque accumulation. In addition to injury severity, future work with larger sample sizes should also examine the role of other injury-related factors, including time since injury, as evidence suggests that longer time since TBI is associated with poorer outcomes,^54,55^ including lower cortical thickness in regions vulnerable to AD,^21^ suggesting a progressive neurodegenerative process following TBI.

Findings of this study also showed that there was no independent effect of PRS on CSF Aβ_42/40_, which was unexpected. However, this is likely due to the specific PRS threshold selected in this study, which was based on the interaction with TBI. Some work suggests that AD may be more oligogenic than polygenic,^56^ which would manifest as a main effect of PRS on AD and AD-related pathology at more stringent PRS thresholds that predominantly include the most penetrant SNPs. Some research has shown a main effect of PRS at stringent,^18^ but not lenient,^21^ thresholds. It is possible that the core genetic pathways of AD may be oligogenic, but environmental factors, such as TBI, may implicate more peripheral genetic pathways of AD, which would only be included in more lenient PRS thresholds, thus contributing to no significant main effects at lenient thresholds but significant interactive effects. Consistent with the notion that the AD phenotype may be oligogenic and characterized by select, penetrant genes, *APOE* ε4 carrier status was a strong predictor of CSF Aβ_42/40_, which is consistent with prior research showing *APOE* ε4 is the greatest genetic risk locus for AD.^16^ *APOE* ε4 carrier status did not interact with TBI, which further supports the hypothesis that TBI may implicate more peripheral genetic pathways of AD. It is also possible that there was a floor effect, whereby *APOE* ε4 carriers had consistently low Aβ_42/40_. Nonetheless, the main effect of *APOE* ε4 underscores the importance of this genetic variant in AD pathogenesis.

This study has several limitations. First, our sample is relatively small, and the findings should be considered provisional until the study can be replicated with larger sample sizes. Moreover, it is imperative to validate these results in additional cohorts. In addition, due to the cross-sectional nature, we cannot determine if participants will eventually progress to AD or if there were preexisting differences in Aβ levels prior to sustaining a TBI. Future work should examine interactions between TBI, PRS, and AD pathology longitudinally. Similarly, while we examined Aβ_42/40_ due to its specificity to AD, it is unknown if the observed amyloid pathology is indicative of AD versus another neurodegenerative disease. Another limitation is that lifetime TBI history was self-reported, which is inherently prone to bias, but common in research and a limitation in many studies.^57^ Finally, our sample only included male Vietnam War Veterans who identified as White non-Hispanic/Latino, and therefore, it is unclear how these results generalize to females, civilians, and other racial/ethnic groups.

## Conclusions

This study suggests that the combination of factors, TBI and polygenic risk for AD, is associated with greater CSF Aβ pathology than each factor alone, although results should be considered provisional until this study can be replicated with larger samples. Specifically, among individuals with a prior TBI, higher AD PRS was associated with lower CSF Aβ_42/40_, suggesting greater amyloid deposition in the brain, while there was no relationship between PRS and Aβ_42/40_ among individuals without a prior TBI. These associations were independent of *APOE* status, although *APOE* ε4 was associated with lower CSF Aβ_42/40_. In addition, findings suggested that relationships between TBI, PRS, and CSF Aβ_42/40_ may be stronger with more severe injuries. These results provide pivotal insight into who may be at increased risk for AD neuropathology following TBI and emphasize the importance of studying the genetic pathways associated with AD risk beyond *APOE* ε4 alone, particularly in the context of TBI.

## Data Availability

All data used in this study are publicly available through the Department of Defense Alzheimer’s Disease Neuroimaging Initiative repository (DOD-ADNI; adni.loni.usc.edu).

## Abbreviations Used

AD: Alzheimer’s disease
ADNI: Alzheimer’s Disease Neuroimaging Initiative
APP: amyloid precursor protein
APOE: apolipoprotein E
Aβ: β-amyloid
CAPS-IV: Clinician Administered PTSD Scale
CSF: cerebrospinal fluid
DOD-ADNI: Department of Defense Alzheimer’s Disease Neuroimaging Initiative
GWAS: genome-wide association study
IRBs: Institutional Review Boards
PRS: polygenic risk score
PTSD: posttraumatic stress disorder
SNPs: single nucleotide polymorphisms
TBI: traumatic brain injury
