# Supplementary material for: Traumatic Brain Injury and Genetic Risk for Alzheimer’s Disease Impact Cerebrospinal Fluid β-Amyloid Levels in Vietnam War Veterans
Source: Neurotrauma Rep. 2024 Aug 22;5(1):760–9. doi: 10.1089/neur.2024.0048 (PMC11342050; doi:10.1089/neur.2024.0048)
Supplement: Supplementary table S1 [file neur.2024.0048_hayesetable1.pdf]

eTable 1. Linear regression results for each polygenic risk score *P*-value threshold.

	**PRS Main Effect**				**PRS x TBI Interaction**			
***P*-value threshold**	***B***	***SE (B)***	$P_{uncorrected}$	$P_{corrected}$	***B***	***SE (B)***	$P_{uncorrected}$	$P_{corrected}$
0.05	-0.203	0.103	0.05	0.10	-0.381	0.203	0.06	0.11
0.10	-0.171	0.104	0.10	0.18	-0.380	0.205	0.07	0.11
0.20	-0.134	0.105	0.21	0.34	-0.362	0.206	0.08	0.14
0.30	-0.128	0.106	0.23	0.37	-0.443	0.205	0.034*	0.058
0.40	-0.116	0.107	0.28	0.43	-0.445	0.205	0.033*	0.056
**0.50**	**-0.118**	**0.107**	**0.27**	**0.42**	**-0.455**	**0.204**	**0.029***	**0.0495***

Results reported are for the main effect of polygenic risk and the interaction between polygenic risk and TBI on $A\beta_{42/40}$. Main effects of PRS are reported from models including covariates and the main effects of TBI and PRS. Interaction effects are reported from models including covariates, the main effects of TBI and PRS, and their interaction. Polygenic risk and $A\beta_{42/40}$ were standardized for analyses. *$P < 0.05$
