# Supplementary material for: Traumatic Brain Injury and Genetic Risk for Alzheimer’s Disease Impact Cerebrospinal Fluid β-Amyloid Levels in Vietnam War Veterans
Source: Neurotrauma Rep. 2024 Aug 22;5(1):760–9. doi: 10.1089/neur.2024.0048 (PMC11342050; doi:10.1089/neur.2024.0048)
Supplement: Supplementary table S2 [file neur.2024.0048_hayesetable2.pdf]

eTable 2. Demographics as a function of most severe TBI.

	**Mild (n = 23)**	**Moderate/Severe (n = 24)**	**Statistics**
**Age in years**	69.6 (3.98)	67.9 (2.72)	$t(38.69)=1.66$, $P=0.11$
**Education in years**	15.0 (2.54)	15.3 (2.42)	$t(44.63)=-0.40$, $P=0.69$
**CAPS-IV severity score**	51.7 (33.0)	41.7 (23.2)	$t(39.28)=1.20$, $P=0.24$
**Polygenic risk score**[a]	0.091 (1.10)	-0.199 (0.86)	$t(41.76)=1.01$, $P=0.32$
***APOE* ε4 status, *n* (%)**[b]			OR=1.83, P=0.50[c]
0	18 (78%)	17 (71%)	
1	4 (17%)	7 (29%)	
**$A\beta_{42/40}$**[a]	0.108 (0.98)	-0.138 (1.03)	$t(45)=0.84$, $P=0.41$
**Number of lifetime TBIs, *n* (%)**			OR=1.31, P=0.77[c]
1	14 (61%)	13 (54%)	
2+	9 (39%)	11 (46%)	

Values presented are mean (SD) unless otherwise indicated. Statistics reported are from *t*-tests unless otherwise indicated. [a]Standardized value. [b]One participant with a mild TBI was missing *APOE* ε4 status data. [c]Statistics reported from Fisher's exact test.
