# Supplementary material for: Traumatic Brain Injury and Genetic Risk for Alzheimer’s Disease Impact Cerebrospinal Fluid β-Amyloid Levels in Vietnam War Veterans
Source: Neurotrauma Rep. 2024 Aug 22;5(1):760–9. doi: 10.1089/neur.2024.0048 (PMC11342050; doi:10.1089/neur.2024.0048)
Supplement: Supplementary table S3 [file neur.2024.0048_hayesetable3.pdf]

eTable 3. Summary of ANCOVA analysis for association between polygenic risk, TBI severity, and $A\beta_{42/40}$.

Variable	*Type III SS*	df	*F*	*P*
Age	0.01	1	0.01	0.93
Education	0.62	1	0.73	0.40
CAPS-IV Score	6.78	1	7.99	0.01*
TBI Severity	1.21	2	0.71	0.49
PRS	0.53	1	0.62	0.43
TBI Severity x PRS	5.11	2	3.01	0.05

Polygenic risk and $A\beta_{42/40}$ were standardized for analyses. PRS threshold is $P<0.50$. *$P<0.05$
