# Supplementary material for: Traumatic Brain Injury and Genetic Risk for Alzheimer’s Disease Impact Cerebrospinal Fluid β-Amyloid Levels in Vietnam War Veterans
Source: Neurotrauma Rep. 2024 Aug 22;5(1):760–9. doi: 10.1089/neur.2024.0048 (PMC11342050; doi:10.1089/neur.2024.0048)
Supplement: Supplementary table S4 [file neur.2024.0048_hayesetable4.pdf]

eTable 4. Summary of regression analysis for association between polygenic risk excluding *APOE*, TBI, and Aβ$_{42/40}$.

**Variable**	**Model 1**			**Model 2**			**Model 3**		
	B	***SE (B)***	***P***	***B***	***SE (B)***	***P***	***B***	***SE (B)***	***P***
Age	0.01	0.03	0.70	0.01	0.03	0.70	0.01	0.03	0.64
Education	-0.05	0.05	0.33	-0.05	0.05	0.29	-0.07	0.05	0.15
CAPS-IV Score	0.01	0.003	0.003*	0.01	0.003	0.006*	0.01	0.003	0.003*
TBI				-0.19	0.21	0.37	-0.18	0.20	0.38
PRS				-0.10	0.11	0.38	0.15	0.15	0.33
TBI x PRS							-0.46	0.20	0.03*
R^2		0.122			0.138			0.189	
Model *F*		3.872*			2.624*			3.145*	

Polygenic risk and Aβ$_{42/40}$ were standardized for analyses. The main effects are reported from model 2. The interaction between TBI and PRS is reported from model 3. PRS threshold is $P<0.50$. $^*P<0.05$
