# Supplementary material for: Traumatic Brain Injury and Genetic Risk for Alzheimer’s Disease Impact Cerebrospinal Fluid β-Amyloid Levels in Vietnam War Veterans
Source: Neurotrauma Rep. 2024 Aug 22;5(1):760–9. doi: 10.1089/neur.2024.0048 (PMC11342050; doi:10.1089/neur.2024.0048)
Supplement: Supplementary table S5 [file neur.2024.0048_hayesetable5.pdf]

eTable 5. Summary of regression analysis for association between *APOE* ε4, TBI, and $A\beta_{42/40}$.

Variable	Model 1			Model 2			Model 3		
	B	***SE (B)***	***P***	***B***	***SE (B)***	***P***	***B***	***SE (B)***	***P***
Age	0.01	0.03	0.70	-0.03	0.03	0.33	-0.03	0.03	0.30
Education	-0.05	0.05	0.33	-0.03	0.04	0.54	-0.02	0.04	0.63
CAPS-IV Score	0.01	0.003	0.004*	0.01	0.003	0.001*	0.01	0.003	0.001*
TBI				-0.11	0.18	0.56	-0.04	0.21	0.85
APOE ε4				-1.12	0.22	<0.001*	-0.96	0.33	0.004*
TBI x *APOE* ε4							-0.28	0.43	0.51
R^2		0.121			0.345			0.349	
Model *F*		3.801*			8.536*			7.136*	

$A\beta_{42/40}$ was standardized for analyses. The main effects are reported from model 2. The interaction between TBI and *APOE* ε4 is reported from model 3. $*P<0.05$
